# Supplementary material for: Extent of Spin Contamination Errors in DFT/Plane-wave Calculation of Surfaces: A Case of Au Atom Aggregation on a MgO Surface
Source: Molecules. 2019 Jan 30;24(3):505. doi: 10.3390/molecules24030505 (PMC6385026; doi:10.3390/molecules24030505)
Supplement: Supplementary file 1 [file molecules-24-00505-s001.zip › Supplementary Materials/TableS1_FigS1_v1.pdf]

Supplementary Materials for

"Effect of spin contamination error and importance of open-shell structure for aggregation of Au atoms on a MgO surface"

Kohei Tada [1,*], Tomohiro Maruyama [2], Hiroaki Koga [3], Mitsutaka Okumura [2,3], and Shingo Tanaka [1]

[1] Department of Energy and Environment, National Institute of Advanced Industrial Science and Technology (AIST), 1-8-31, Midorigaoka, Ikeda, Osaka, 563-8577, Japan

[2] Department of Chemistry, Graduate School of Science, Osaka University,1-1, Machikaneyama, Toyonaka, Osaka, 560-0043, Japan

[3] Elements Strategy Initiative for Catalysts and Batteries (ESICB), Kyoto University, 1-30 Goryo Ohara, Nishikyo, Kyoto, Kyoto 615-8245, Japan

* Corresponding Author

phone: +81-72-751-8566

fax: +81-72-751-9714

k-tada@aist.go.jp

Contents

- Table S1 p. 3
- Figure S1 p. 4

Captions for Table S1 and Figure S1

Table S1 The nearest neighbor atoms of the ^{2}Au atom for all r.

Figure S1. Spin density distributions of the HS states. Isosurface values are 0.004 e/Bohr^3.

Table S1 The nearest neighbor atoms of the ^{2}Au atom for all r.

r	LS	HS	BS
0.000	^{1}Au	^{1}Au	^{1}Au
0.025	^{1}Au	^{1}Au	^{1}Au
0.050	^{1}Au	^{1}Au	^{1}Au
0.075	^{1}Au	^{1}Au	^{1}Au
0.100	^{1}Au	^{1}Au	^{1}Au
0.125	^{1}Au	^{1}Au	^{1}Au
0.150	^{1}Au	^{1}Au	^{1}Au
0.175	^{1}Mg	^{1}Mg	^{1}Mg
0.200	^{2}O	^{2}O	^{2}O
0.225	^{2}O	^{2}O	^{2}O
0.250	^{2}O	^{2}O	^{2}O
0.275	^{2}O	^{2}O	^{2}O
0.300	^{2}O	^{2}O	^{2}O
0.325	^{2}Mg	^{2}Mg	^{2}Mg
0.350	^{2}Mg	^{2}Mg	^{2}Mg
0.375	^{2}Mg	^{2}Mg	^{2}Mg
0.400	^{2}Mg	^{2}Mg	^{2}Mg
0.425	^{2}Mg	^{2}Mg	^{2}Mg
0.450	^{3}O	^{3}O	^{3}O
0.475	^{3}O	^{3}O	^{3}O
0.500	^{3}O	^{3}O	^{3}O

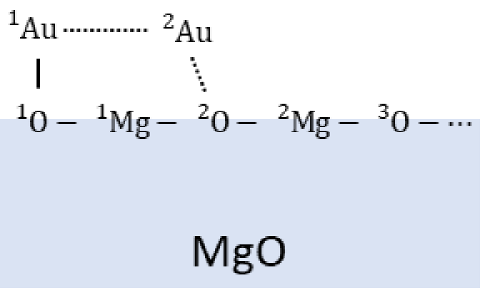

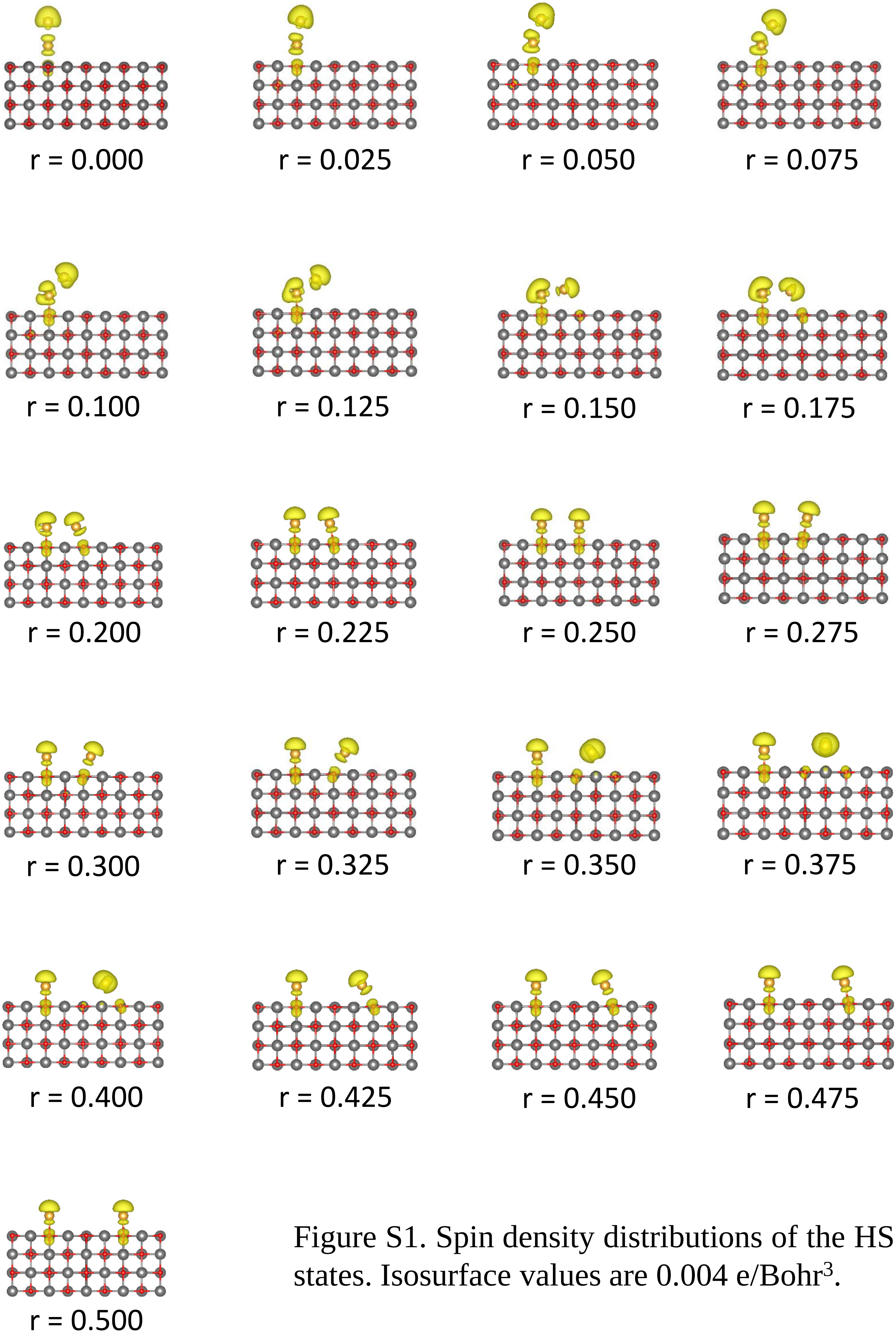

Figure S1. Spin density distributions of the HS states. Isosurface values are 0.004 e/Bohr^3.
